# Metabolic landscapes in sarcomas

**DOI:** 10.1186/s13045-021-01125-y

**Published:** 2021-07-22

**Authors:** Richard Miallot, Franck Galland, Virginie Millet, Jean-Yves Blay, Philippe Naquet

**Affiliations:** 1 Centre National de la Recherche Scientifique, Institut National de la Santé et de la Recherche Médicale, Centre d’Immunologie de Marseille Luminy, Aix Marseille Univ, Marseille, France; 2 Centre Léon Bérard, Lyon 1, Lyon Recherche Innovation contre le Cancer, Université Claude Bernard, Lyon, France

**Keywords:** Sarcoma, Metabolism, Microenvironment, Metabolomics, Transcriptomics, Metabolite-targeted therapies

## Abstract

Metabolic rewiring offers novel therapeutic opportunities in cancer. Until recently, there was scant information regarding soft tissue sarcomas, due to their heterogeneous tissue origin, histological definition and underlying genetic history. Novel large-scale genomic and metabolomics approaches are now helping stratify their physiopathology. In this review, we show how various genetic alterations skew activation pathways and orient metabolic rewiring in sarcomas. We provide an update on the contribution of newly described mechanisms of metabolic regulation. We underscore mechanisms that are relevant to sarcomagenesis or shared with other cancers. We then discuss how diverse metabolic landscapes condition the tumor microenvironment, anti-sarcoma immune responses and prognosis. Finally, we review current attempts to control sarcoma growth using metabolite-targeting drugs.

## Background

Sarcomas encompass a wide variety of tumors, with more than 170 subtypes, according to the last WHO classification. They originate from the neoplastic transformation of mesenchymal cells in connective tissues [1, 2]: 87% arise from soft tissue and 13% from bone [3, 4]. Soft tissue sarcoma (STS) presents as an indolent or aggressive disease, often only diagnosed at an advanced and/or metastatic stages. Current sarcoma classification relies on histopathology that may lead to errors in up to a quarter of cases [5]. In terms of prevalence, they represent less than 1% of adult cancers, but up to one fifth of pediatric solid malignant cancers [3]. Surgery is the standard of care for patients supplemented with chemotherapy or radiotherapy [6]. Targeted therapies remain limited to tumors with well-defined oncogenic drivers [1, 2]. Clinical trials targeting immune checkpoints show low response rates, with few responsive histotypes. Finally, biomarkers or tertiary lymphoid structures may be be predictive tools for 10% of patients [7]. Consequently, improving sarcoma typing and treatment requires the use of large-scale “omics” tools to identify the oncogenic drivers, often resulting from multiple genetic alterations in adult STS. These can include translocations, mutations or amplifications/deletions that cripple major growth and differentiation pathways [8–12].

Given the limits of current treatments, exploiting drugs targeting metabolic pathways may pave the way to effective therapy for these largely incurable diseases.

Aggressive tumors must survive in a reorganized, stressful and metabolically competitive microenvironment. This necessary adaptation exploits tumor heterogeneity and cell networks in the tumor microenvironment. Furthermore, within a given cell, plasticity depends on interconnections between various metabolic pathways to adapt growth to the available metabolites. A major trait often amplified in these tumors is the use of aerobic glycolysis, known as the Warburg effect [13], that optimizes tumor cell growth through provision of building blocks to increase biomass [14]. Since Warburg’s discovery, a debate has existed about the persistence of mitochondrial activity in glycolytic tumors and its potential to be a drug target [15]. Despite the central role of mitochondria not only in cell energetics, homeostasis and stress sensing [16] but also reactive oxygen species (ROS) production [17] their contribution to oncogenic transformation is still debated. In some STS, germline mutations affecting mitochondrial enzymes lead to the accumulation of oncometabolites that induce a pseudo-hypoxic response and alter epigenetic marks and differentiation [18]. In the tumor microenvironment, glycolytic and oxidizing cells may compete or cooperate for an optimal use and exchange of energetic metabolites. This network involves immune cells that adapt their metabolism to exert their functions in this competitive environment [19]. The purpose of this review is to link recent findings on STS genetics to the alterations of intracellular pathways affecting their tumor metabolic landscapes. Although not necessarily specific to STS, they may represent novel therapeutic opportunities.

### Unsupervised omics and single cell-based analyses highlight metabolic signatures in cancer

The development of more integrated technologies with increased sensitivity and/or resolution has helped to unravel tumor genomic and metabolic complexity in situ and to bridge the gap between mouse models and patients. Several recent studies documented the power of integrated genomic or metabolomic strategies to decipher tumors complexity. An article from The Cancer Genome Atlas (TCGA) Research Network [8] combined genetic, epigenetic and transcriptomic analyses and proposed a novel classification of STS subtypes with complex genomes. In their analysis of the number and nature of copy number variations (CNVs), they identified three dominant profiles from leiomyosarcoma (LMS), myxofibrosarcoma (MFS), undifferentiated pleomorphic sarcoma (UPS) to dedifferentiated liposarcoma (DDLPS) displaying the highest level of genomic alterations. In addition to these modifications, the nature of epigenetics marks, activating pathways or immune signatures add further prognostic value. Another article exploited TCGA data to describe the relative contribution of 114 metabolic pathways to cancer progression [20]. This analysis showed that master metabolic transcriptional regulators behave as genetic drivers explaining the metabolic profiles displayed by various tumors compared to normal tissues, and help predict responsiveness to metabolism-targeting drugs. For example, alterations of specific transcriptional regulators explain the defect in polyamine biosynthesis in prostate cancer. Similarly, distinct pathways enriched in breast cancer allow the discrimination of aggressive tumors from those associated with a good prognostic. Based on this finding, metformin, a mitochondrial complex 1 inhibitor, has been proposed as a potential adjunct therapy against basal breast cancer cells, due to its unique deregulation of the Tricarboxylic Acid (TCA) cycle. In STS, this analysis highlighted the enrichment in the pentose and glucuronate interconversion (PGI) pathway, also amplified in the Yang Huang syndrome described in the context of traditional Chinese pharmacology [21]. The PGI pathway relies on UDP-glucuronosyltransferase (UGT) enzymes that catalyze the binding of d-glucuronic acid to toxic substances or endogenous compounds such as bilirubin via glycosidic bonds, contributing to the detoxification of lipophilic compounds or glucuronides.

**Fig. 1:**
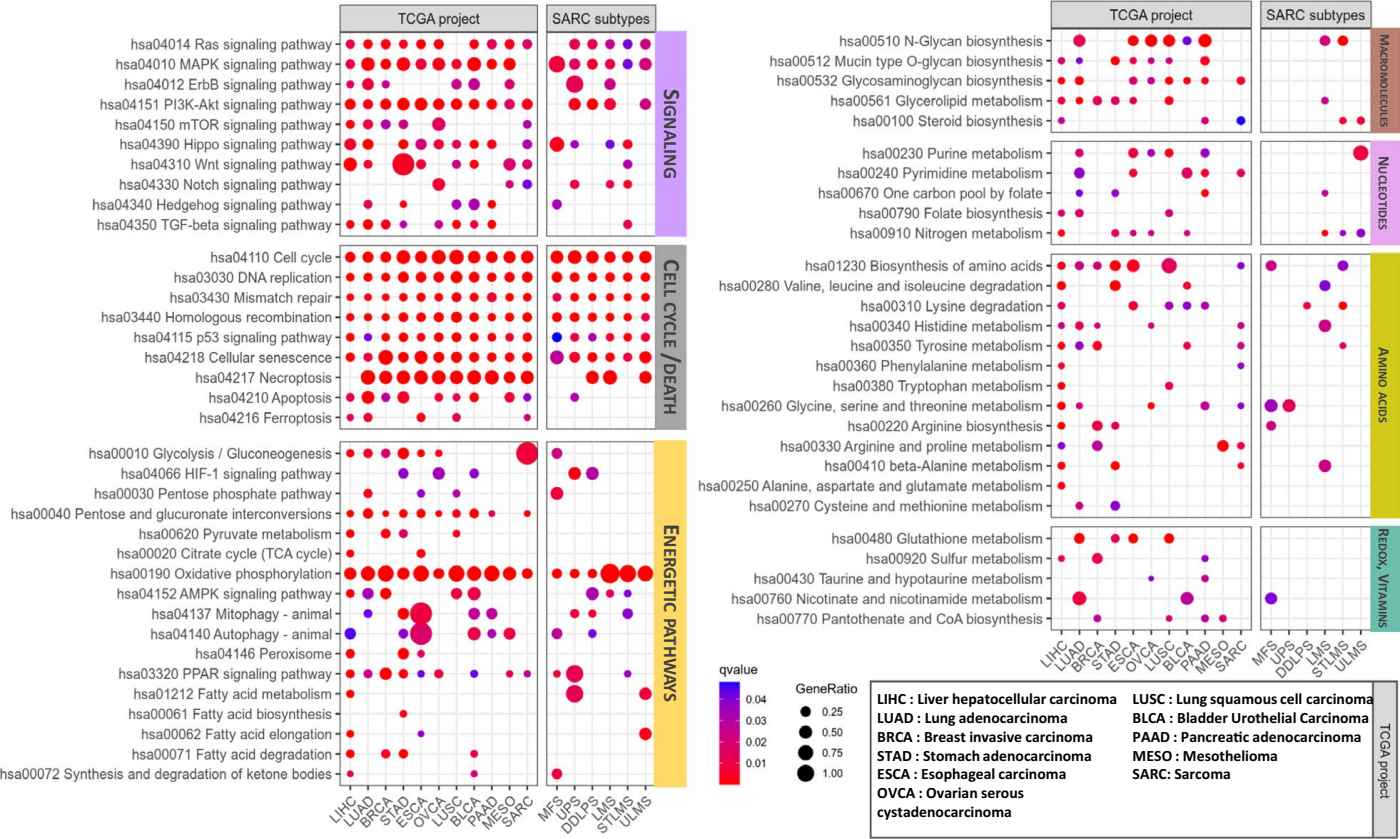
Analysis of the TCGA transcriptomic database. Dotplots showing functional enrichment for co-expression modules found in various cancer types and predominant sarcoma subtypes. Htseq raw counts were retrieved from TCGA using GDCquery [22] and VST-normalized [23]. For each dataset, the unsigned co-expression network was produced using WGCNA with automatic pick for soft-thresholding powers. Genes in each module were queried for functional enrichment against Reactome Pathway Database [24] using clusterProfiler [25]. *p* values were adjusted using Benjamini–Hochberg procedure. For each dataset-pathway pair, the *p* value corresponds to the lowest one from all the co-expression modules. A subset of the significant (*q*-value < 0.05) pathways was manually annotated into functional groups for display in the figure. Dots highlight significant pathway-dataset pairs

Exploration of the TCGA database allows one to identify more discrete signatures displayed by major STS subtypes versus other types of cancers. As shown in Fig. 1, all cancer types display abnormalities in cell cycle regulation. Most carcinomas show an enrichment in oncogenic pathways, glycolytic signatures and alterations of energetic, nucleotide, amino acid or macromolecule pathways. When considering STS as a whole, RAS, PI3K and HIPPO pathways light up, as in [8], coupled to a dominant glycolytic/OXPHOS signature. More discrete signals confirm the enhancement of the PGI pathway in STS, although this trend is not detectable when considering individually the STS subtypes. Our analysis also indicates that distinct signatures preferentially match with STS subtypes, with UPS featuring an enrichment in PPAR/fatty acids and glycine/serine/threonine pathways, whereas LMS display an enhanced OXPHOS signature. Similarly, differences in oncogenic pathway usage are apparent but it is difficult to relate these pathways to the metabolic bias in tumors.

The improvement of chromatographic and mass spectrometry (MS) analyses such as Ultra High Performance Liquid Chromatography Q-Exactive MS (UHPLC-QE MS) has allowed time to be saved in sample separation while preserving the detection capacity of a large spectrum of metabolites. In osteosarcoma (OS), studies combining state-of-the-art transcriptomic and metabolomics approaches highlighted the amplification of nucleotide and amino acid (namely alanine, aspartate, glutamate, arginine, proline, methionine) pathways, glycolysis and the pentose phosphate shunt [26, 27]. Spatially-correlated analysis, mass spectrometry imaging (MALDI-MSI) can further reveal how biomolecular ions are distributed on tissue sections, linking their molecular identification to their spatial distribution. Results from two studies [28, 29] comparing STS subtypes showed that the overexpression of acyl-CoA-binding protein and stearyl-CoA desaturase (directly involved in the processing of fatty acids) as well as MIF1, galectin 1, thioredoxin could help distinguish LMS from MFS, and predict their prognostic. To identify tumor-associated metabolites in situ, airflow-assisted desorption electrospray ionization mass spectrometry imaging (AFADESI-MSI) was used on tissues from 256 esophageal cancer (ESCA) patients [30]. This analysis unraveled the dysregulation of several metabolic pathways affecting proline, glutamine, histidine, uridine, fatty acid and polyamine homeostasis. Among others, it identified pyrroline-5-carboxylate reductase 2 (PYCR2) and ornithine decarboxylase (ODC), rate-limiting enzymes in proline and polyamine biosynthesis, respectively, as markers of tumor proliferation. Table 1 summarizes biomarkers and metabolites linked to alterations in the activation of metabolic pathways in STS. In the future, these explorations will benefit from single-cell strategies evaluating the metabolic status of tumor versus surrounding cells. In this context, the development of novel flow cytometry-based methods to assess metabolic activity such as Met-Flow [31] or SCENITH [32] have already proven their potential to assay the metabolic status of circulating or tumor-infiltrating immunocytes.

**Table 1:** Biomarkers and metabolites associated with STS

	Cell processes	Biomarkers (genes or metabolites)	STS subtype	Prognosis significance	References
Signaling	RAS signaling	GLUT, HK, PFK	UPS, MFS	Poor prognosis	[33, 34]
	PI3K-AKT signaling		LMS, EWS	Poor prognosis	[35, 36]
		miR-181b	STLMS, ULMS	RFS	[8]
		MDM2 amplification	DDLPS	Poor prognosis	
	GFR signaling	IGFR1 overexpression	STLMS, EWS, MLS, ARMS, SS	Poor RFS/DSS	[8, 37, 38]
		Her4/Erbb4	OS, EWS	Poor prognosis	[38]
		Serum bFGF, VEGF	STS	Poor prognosis	[39]
	JUN signaling		DDLPS	Poor prognosis	[8]
	HIPPO pathway	Nuclear YAP/TAZ, VGLL3	UPS, MFS, MLS, RMS	Poor prognosis	[40–45]
	WNT pathway	Nuclear β-catenin/LEF1; MEG3 (lncRNA) downregulation	EWS, OS	Poor prognosis	[46, 47]
Cell cycle/death	Cell cycle	CINSARC—67 genes	STS	Poor prognosis	[48]
		TP53, RB1, CDKN2A deficiency	LMS, UPS, MFS	Poor prognosis	[8, 49–51]
		TP53, IGAR, GLUT	LMS, UPS, MFS, EWS	Poor prognosis	
		CDCA2, KIF14, IGBP7	SS	Metastasis	[52]
		CDK4 amplification	DDLPS	Poor prognosis	[8]
	DNA replication	TOP2A	MPNST	Poor prognosis	[8]
		RRM1	OS, EWS	Good prognosis	[8]
		ATRX deletion	DDLPS	Poor prognosis	[8]
	Transcriptional regulation	DNA hypermethylation,	DDLPS, STLMS	poor RFS/DSS	[8]
		HMGA2 amplification	DDLPS	Poor prognosis	[8]
Energetic pathways	Glycolysis	GLUT, ENO1, TPI1, PKG1, LDHC, lactate, pyruvate	STS	Poor prognosis	[20, 53, 54]
		*FBP2 loss*	LPS, LMS, FS, UPS	Poor DSS	[55]
		*Serum LDH*	ULMS, EWS	Poor prognosis	[39, 51]
		*PKM1/2 isoenzymes*	OS	Poor prognosis	[56]
	Pentose and glucoronate interconversions	UGT	STS	Prognosis	[20]
	Citrate cycle/OXPHOS	Downregulated metabolites	OS	Poor prognosis	[27]
		Decreased ATP Synthase subunits	OS	Poor prognosis	[56]
		SDH, FH mutations (succinate accumulation)	GIST	Poor prognosis	[57]
	Others	AMPKa, CHK1, S6, ARID1A, RBM15, MSH6, AcetylTubulin	STS	Combined survival related signature	[58]
	Nucleotide metabolism		STS	Poor prognosis	[57]
Amino acids	Alanine, aspartate, glutamate	GLS	OS, KS, EWS	High risk	[27, 59, 60, 56]
	Arginine, ornithine	ASS1 deficiency, ODC	OS, MFS, KS	DSS, MFS	[27, 61, 62]
	Proline	PYCR2	OS, KS	DSS, MFS	[27, 63]
	Serine, glycine	PHGDH, PSAT1, PSPH, SHMT2, SLC1A5, MTHFD2, MTHFD1L	EWS	DSS, MFS	[60, 64]
	Tryptophane	TDO2 (low)	EWS	DSS, MFS	[65]
	5 methylthioadenosine		OS	DSS, MFS	[27]
Redox, vitamins	Pantothenate metabolism	VNN1 (low)	FS	Poor prognosis	[66]
	Redox metabolism	TXR, MIF1, GAL1, AcCoaBP	LMS (high), MFS (low)	Poor prognosis	[67]
	Hypoxia	HIF1α, hypoxia gene signatures	EWS, OS, GIST, KS	Poor prognosis	[68–72]

Here, we will review and update the mechanisms that link complex oncogenic stimuli associated with various STS to metabolic alterations.

### Oncogenic drivers upstream of growth pathways rewire metabolism in sarcomas

**Fig. 2:**
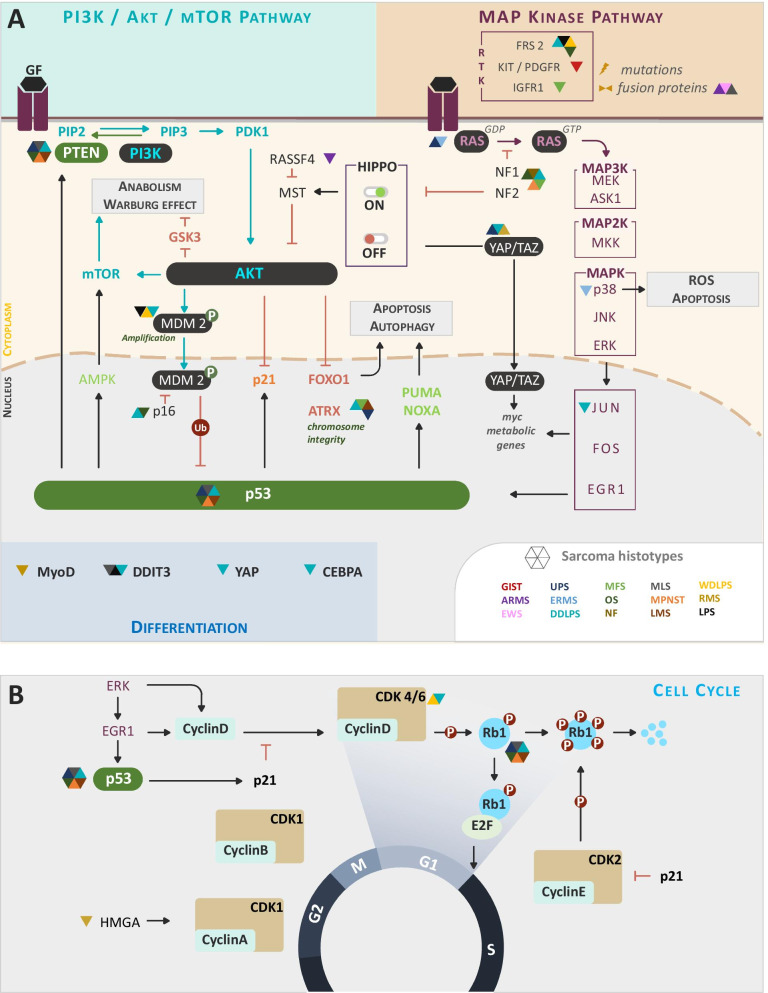
Oncogenic and tumor suppressor pathways altered in STS. (**A**) This figure highlights mutations that alter regulations of PI3K/AKT/mTOR and MAP kinase pathways in sarcoma. Colored triangles associate sarcoma subtypes (listed on the bottom right corner) with the corresponding genes alterations, either expression or loss, on the scheme. Expression or regulations of tumor suppressor genes is altered (p53, PTEN) concomitantly with increased expression of oncogenes driving malignant transformation (increase Anabolism, Warburg effect). (**B**) Panel B focuses on cell cycle alterations at the level of the p53 and RB1 tumor suppressor genes notably

**Fig. 3:**
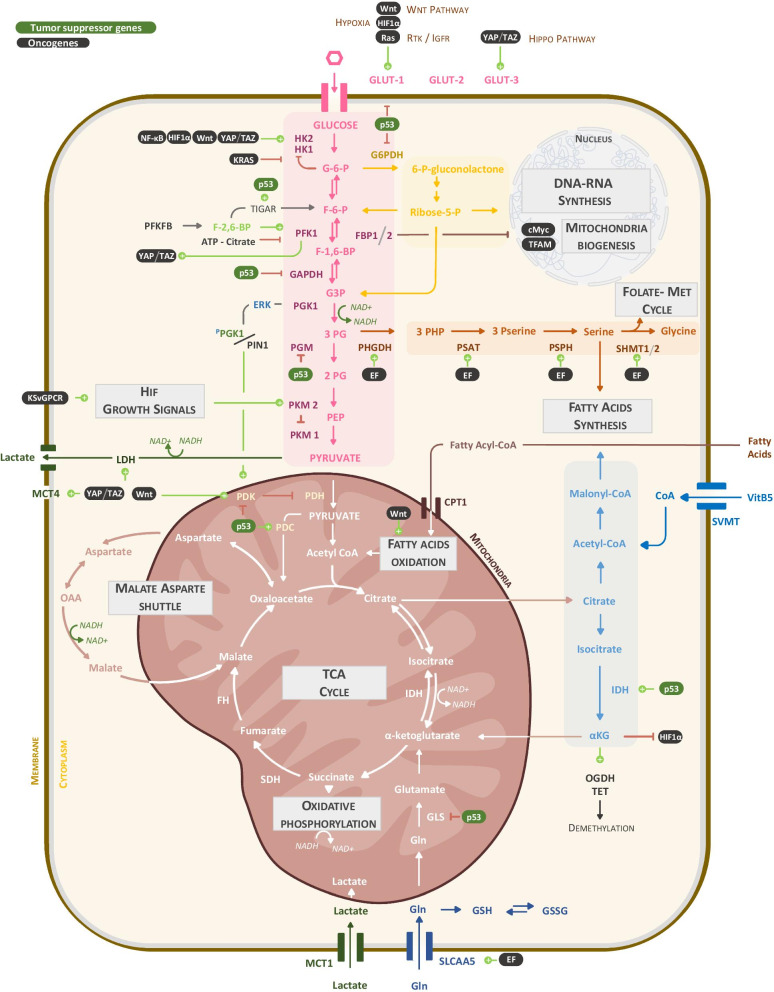
Metabolic consequences of STS-associated molecular alterations. This scheme integrates sarcoma genetic alterations affecting tumor suppressor genes (green background) or oncogenes (black background) in the tumor metabolic network. These alterations enhance enzymatic reactions in favor of anabolic pathways by increasing the glycolytic flux (pink) and branched pathways, notably nucleotide (yellow), fatty acids (orange) and DNA/RNA synthesis at the cost of dampens mitochondrial function and TCA cycle proper functioning

Physiologically, growth factor receptors (GFR) trigger the RAS/MAPK and PI3K/AKT/mTOR pathways and activate transcriptional regulators such as JUN/FOS/EGR1 that drive cell division (Fig. 2A). This process is temporally regulated by the co-engagement of restriction points controlled by tumor suppressor genes (TSGs) (Fig. 2B) [73]. In cancer cells, prolonged exposure to oncogenic signals strongly stimulates ERK-dependent EGR1 activation, bypassing cell cycle regulation and provoking PI3K activation that antagonizes p53-dependent tumor suppression [74] (Fig. 2B). In p53-mutated cancers, the temporal regulation of MEK/MYC/PI3K is dysfunctional and this allows cancer cells exposed to transient growth signals to proliferate, in a context of increased genomic instability. These pathways have been shown to be involved in sarcomagenesis in both human and rodent models (Fig. 2A), downstream of oncogenic GFRs or receptors involved in tissue organization and trophicity. We will highlight how various activation pathways engage these metabolic programs to sustain STS cell growth and rely alternatively on various carbon sources such as glucose, amino acids or lipids (Fig. 3). We also provide an update on current clinical trials exploiting metabolic interference in Table 2.

#### Overactivated MAP and PI3 kinase pathways drive a Warburg effect

Both mutations and oncogene-driven overexpression of GFRs contribute to STS development. Gain-of-function mutations of the GFR KIT or PDGF-Rα drive gastrointestinal stromal tumor (GIST) progression [93]. In the translocation-associated Ewing’s sarcoma (EWS), myxoid liposarcomas (MLS) or alveolar rhabdomyosarcoma (ARMS), the fusion proteins EF, FUS-DDIT3 or PAX3-FOXO1, respectively, enhance IGF1R expression, a major driver of RAS/AKT/mTOR activation [37]. Hyperactivation of the RAS pathway is predictive of a high risk of disease recurrence and impaired overall survival in 30% undifferentiated pleomorphic sarcoma (UPS), a common adult STS [33, 34]. Similarly, the loss of the phosphatase PTEN induces growth-factor independent PI3K/AKT activation that sustains autonomous nutrient uptake in some LMS or MPNST [94]. Accordingly, sarcoma incidence increases in hereditary neurofibromatosis patients with carrying deletions of the RAS negative regulator genes NF1 or NF2 [49, 95]. To investigate the mechanisms of tumor progression in a mouse model, whole-exome sequencing was performed on STS induced by either KrasG12D activation/p53 deletion, 3-methylcholanthrene (MCA) or ionizing radiation [96]. Whereas CNVs were very frequent in radiation-induced STS, MCA-induced tumors showed a high mutational burden, combined with high genomic instability in the absence of p53. Candidate oncogenic drivers affecting MAPK signaling were identified either as mutations of Kras, Nf1 and Hippo effectors (Fat1/4), or as amplification of Kras and Myc in p53 deficient mice, or Met and Yap1 in radiation-induced STS. Mutations in the RAS pathway influence the prognosis of human STS such as DDLPS or pediatric embryonic rhabdomyosarcoma (ERMS) [97]. In the latter, the overactivation of p38 MAPK induces high levels of reactive oxygen species (ROS) that increase the mutation rate [97] and may sensitize tumors to therapies enhancing oxidative stress [98, 99].

**Fig. 4:**
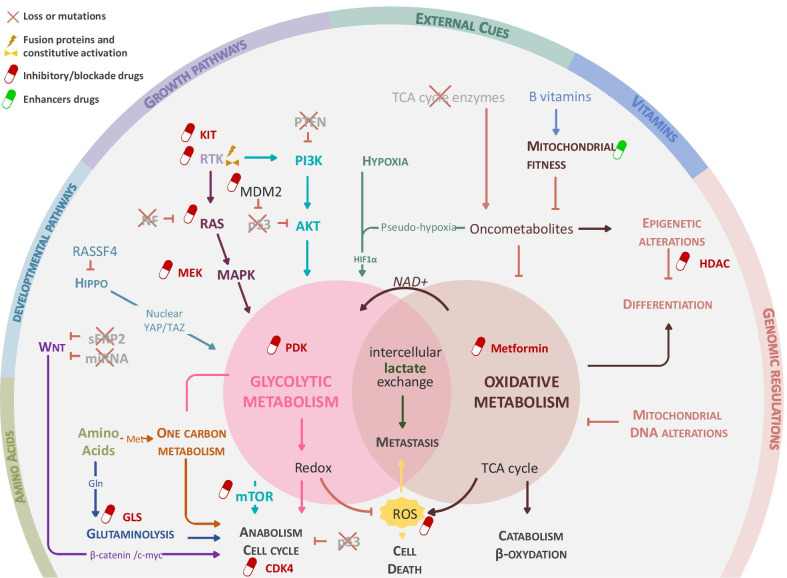
Integrated view of cues and pathways amenable to pharmacological modulation in STS. This diagram places the different existing therapies in sarcoma according to their therapeutic targets. Panel (**A**) stratify therapeutic option according to main cellular pathways and table B index the current clinical trial and biomarker available in sarcoma disease

RAS- or PI3K/AKT-driven activation increases the expression of glucose importers (GLUT) and of the upstream ATP-consuming glycolytic enzymes hexokinase (HK) and phosphofructokinase (PFK), recently shown to control the glycolytic flow quantitatively [100]. The oncogenic KRAS4A isoform and to lesser extent other RAS isoforms were shown to interact with HK1 on mitochondria (Fig. 3), preventing its allosteric inhibition by glucose-6-phosphate (G6P), thereby enhancing glycolysis [101]. Hypoxia or mutations affecting the RAS pathway modulated the activity of PKM2 or PGK1, two ATP-generating enzymes in the last steps of glycolysis, thus providing them with non-metabolic pro-oncogenic functions [102]. Thus, an increase in the proportion of PKM2 dimers, lacking pyruvate kinase activity, drives PKM2 nuclear translocation where it participates in STAT3 phosphorylation and *mek5* gene transcription, driving cell growth [103]. Another study showed that activated ERK phosphorylates PGK1, promoting its association with PIN1 and its translocation into the mitochondria. There, it phosphorylates and activates pyruvate dehydrogenase kinase 1 (PDK1), an inhibitor of pyruvate dehydrogenase (PDH), the checkpoint of pyruvate entry in the TCA cycle [104] (Fig. 3). Globally, these RAS-driven effects reinforce glycolysis over mitochondrial respiration and favor glucose- and glutamine-dependent anabolism as shown in a pancreatic ductal adenocarcinoma (PDAC) model [105, 106]. Several clinical trials are currently based on drugs inhibiting PI3K, AKT, mTOR and ERK signaling in STS (Fig. 4 and Table 2).

**Table 2:** Clinical trials affecting metabolic pathways in STS

	Biomarker target	Therapeutic agent	Tumor type	Biomarker relevance/clinical trial phase	N° Clinical trial	References
MAPK pathways	RAF	Dabrafenib	Advanced solid tumors with BRAF mutations	Phase II	NCT02465060	[75] [A] [B]
		Vemurafenib	Relapsed or refractory advanced solid tumors with BRAF V600 mutations	Phase II	NCT03220035	
		Dabrafenib + trametinib	MULTISARC	Phase III	NCT03784014	
		Dabrafenib + trametinib	BRAF V600E- mutated rare cancers	Phase II	NCT02034110	
	MEK1/2	Binimetinib + pexidartinib	Advanced GIST	Phase I completed	NCT03158103	[A] [B] [C]
		Trametinib	Advanced solid tumors with BRAF mutations	Phase II	NCT02465060	
		Cobimetinib + MPDL3280A	Locally advanced or metastatic solid tumors	Phase I	NCT01988896	
		GDC-0941 + GDC-0973	Locally advanced or metastatic solid tumors	Phase II	NCT00996892	
	ERK1/2	Ulixertinib	STS, OS, EWS	Phase I/II	NCT03520075	[C]
PI3K/AKT/mTOR signaling	PIK3CA/mTOR	Samotolisib	STS GIST	Phase I/II	NCT02008019	[C] [76]
			Pediatric sarcoma	Phase II MATCH trial	NCT03458728 NCI MATCH EAY131-Z1F	[77] [78]
		GDC-0941	Locally advanced or metastatic solid tumors	Phase I	NCT00876109	[A]
		GDC-0980	Locally advanced or metastatic solid tumors Refractory solid tumors	Phase I	NCT00876122NCT00854152 NCT00854126	[A]
	AKT/ERK	ONC201	Desmoplastic small round cell tumor	In vitro		[C]
		GDC-0973 + GDC-0068	Locally advanced or metastatic solid tumors	Phase I	NCT01562275	[A]
	mTOR	Sirolimus + pexidartinib	STS MPNST	Phase I/II	NCT02584647	[79] [80] [A]
		Rapamycin + gemcitabine	OS	Phase II completed	NCT02429973	
		nanoparticle albumin-bound rapamycin + pazopanib	Advanced nonadipocytic soft tissue sarcomas	Phase I/II trial	NCT03660930	
		Lenvatinib + everolimus	Refractory pediatric solid tumors	Phase I/II	NCT03245151	
		CCI-779	STS/GIST	Phase II	NCT00087074	
		Cixutumumab + temsirolimus	Locally advanced, metastatic, or recurrent STS or bone sarcoma	Phase II	NCT01016015	
		CP-751,871 + RAD001	Advanced sarcomas and other malignant neoplasms	Phase I	NCT00927966	
		Everolimus	RAD001/progressive sarcoma	Phase II	NCT00767819	
HIPPO	YAP/TAZ	Verteporfin	High histological grade	Reduced EWS metastatic potential		[81] [82] [83] [84]
TCA CYCLE	IDH 1	IDH 1—AG-120	Chondrosarcoma	Phase I	NCT02073994	[85] [86] [87]
		IDH 1—FT-2102	Advanced solid tumors	Active	NCT03684811	
		IDH 1—IDH305	Advanced malignancies with IDH1R132 mutations	Phase I	NCT02381886	
		IDH 1—BAY1436032	IDH1-mutant advanced solid tumors	Active	NCT02746081	
		AG-881	Advanced solid tumors with an IDH1 and/or IDH2 mutation	Phase I	NCT02481154	
		AG-120 + nivolumab	IDH1 mutant tumors	Phase II	NCT04056910	
	IDH2	IDH 2—AG-221	Advanced solid tumors	Phase I/II	NCT02273739	
	TCA cycle enzymes	Devimistat	STS	FDA orphan drug designation		[D]
Amino acids	ASS1 deficiency	ADI-PEG20 + gemcitabine + docetaxel	STS, OS, EWS	Phase II	NCT03449901	[88] [89]
	PDK	DCA	FS	Mice		[90]
	GLS	CB-839—glutaminase inhibitor	GIST	Phase I completed	NCT02071862	[59]
		Telaglenastat	NF1 mutation positive MPNST	Phase II	NCT03872427	
		Telaglenastat + talazoparib	Solid tumors	Phase I + phase II	NCT03875313	
	Heparan sulfate proteoglycans	Sulfen	EWS	Zebrafish model		[91]
	NAMPT	FK866—MV87 inhibitors	FS	Mice		[92]
	Folate receptor α	Pemetrexed	STS	Phase II	NCT04605770	[C]
	Lipid metabolism	CPI-613	CCS	Phase II	NCT01832857	[A] [E]

[A] The Life Raft Group. Gisttrials. https://gisttrials.org/iLRG/showfirstline.php. Accessed 16 June 2021

[B] NIH U.S. National Library of Medicine. Clinicalstrial.gov. https://clinicaltrials.gov/ct2/home. Accessed 16 June 2021

[C] NIH. Cancer.gov. https://www.cancer.gov/about-cancer/treatment/clinical-trials/search/r?loc=0&q=sarcoma&rl=1. Accessed 16 June 2021

[D] Rafael Pharmaceuticals, Inc. https://rafaelpharma.com/research-and-development/cpi-613-drug. Accessed 16 June 2021

[E] ICH GCP. Good Clinical Practice Network. https://ichgcp.net/clinical-trials-registry/NCT04593758. Accessed 16 June 2021

#### An altered HIPPO pathway induces aerobic glycolysis in STS

Several sarcoma histiotypes re-express genes involved in developmental pathways [107–110], such as HIPPO that controls organ size. Its engagement in intercellular adhesion complexes activates the MST and LATS kinases that phosphorylate the transcriptional factors YAP1 and TAZ, promoting their degradation. In contrast, upon nuclear translocation, YAP/TAZ cooperate with mitogenic effectors and boost proliferation (Fig. 2A). HIPPO interferes with the RAS and PI3K pathways that control cell death induction [111], acting as a tumor suppressor pathway [112]. Through cross-inhibition, MST and AKT differentially regulate the expression level of pro-apoptotic effectors (NOXA, FASL, BIM, TRAIL). In addition, ERK induces the expression of anti-apoptotic effectors (BCL2, BCL-XL, IAP, MCL1) partly via YAP/TAZ activation. Furthermore, in a PDAC model, YAP1 amplification can bypass the need for oncogenic KRAS activation [113]. The amount of translocated YAP/TAZ determines their co-activating potential for TEAD transcriptions factors and thereby the balance of proliferation versus cell death [40].

Loss of MST/LATS or YAP overexpression is pro-tumoral in mice [114, 115], and this pathway is often affected in MCA- or radiation-induced STS [96] and UPS [8]. In transgenic models, altering HIPPO effectors alone or in combination with other deficits augmented STS frequency [41]. An increased YAP/TAZ nuclear staining is predictive of poor survival in UPS, DDLPS and ERMS [42–45, 116]. Two studies investigated how fusion gene products mediate sarcomagenesis through alteration of the HIPPO pathway in mice. One study explored MLS that account for 5–10% STS, among which 90% tumors depend on the product of the FUS:DDIT3 translocation [44]. The authors performed a large-scale RNA interference screen and identified YAP1 as a non-redundant oncogenic driver. In MLS cell lines, FUS:DDIT3 led to a two to threefold increase in expression and co-transcriptional activity of YAP1. Co-immunoprecipitation and immunofluorescence studies revealed a physical interaction of YAP1 with FUS:DDIT3 in the nucleus. Pharmacological inhibition of YAP1 activity inhibited the growth of MLS xenografts. The other study, based on a new transgenic model, showed that doxycycline (DOX)-induced expression of YAP1 in the myogenic MYOD1 cell lineage provoked the development of ERMS through the transformation of activated satellite cells [45]. Retrieval of DOX released a YAP1-dependent differentiation block and reduced tumor formation. Transcriptional profiling of the tumors revealed that YAP1 induces pro-oncogenic effector genes and represses terminal differentiation of myoblasts. In line with these observations, an independent study found no evidence that mutant RAS isoforms were responsible for YAP overexpression in myoblasts [116]. In ARMS, the translocation product PAX3-FOXO1 suppresses HIPPO signaling through overexpression of RASSF4, which inhibits the MST1 kinase. Similarly, this effect is linked to PAX3-FOXO1 co-localization with YAP1 in the nuclei of cancer cells. This chimeric transcription factor cooperates with YAP1/TEAD to induce downstream effectors that trigger IGFs and NF-κB activation, and repress senescence and apoptosis in mesenchymal cells [40, 44]. Therefore, mutations of HIPPO effectors can be oncogenic in STS.

The HIPPO pathway restricts tissue growth and is connected to nutrient cues [117, 118]. Upon glucose starvation, AMPK- and LATS-kinases phosphorylate YAP resulting in its degradation [119]. Furthermore, Wang et al. showed that the phosphorylation level of YAP1 at position S61 is regulated by AMPK, itself recruited to YAP protein complexes in the cytosol of glucose-deprived cells. Addition of glucose was associated with a decrease in YAP phosphorylation and its nuclear translocation, where it interacted with TEAD transcriptional regulators to induce glycolytic genes. This glucose-sensing pathway via YAP and TAZ was required for the full deployment of glucose growth-promoting activity in breast cancer. In addition, glycolysis was required to sustain YAP/TAZ tumorigenic properties [120]. Mechanistically, phosphofructokinase (PFK1) bound to and co-activated the YAP/TAZ transcriptional cofactors TEADs (Fig. 3). In some cancers, the loss of NF2, an upstream negative regulator of HIPPO signaling, simultaneously unleashed YAP/TAZ and SMAD2/3 activation leading indirectly to the induction of aerobic glycolysis via derepression of *GLUT*, *HK2*, *LDH* and *MCT* genes [121]. Interestingly, *NF2* mutations might contribute to the maintenance of rare aggressive sarcomas [122]. This hypothesis was tested in a kidney cancer cell model bearing NF2 mutations [123]. There, DOX-induced expression of shRNA downregulating YAP/TAZ expression provoked the regression of tumors in vivo. YAP/TAZ-depletion induced a substantial decrease in EGFR and AKT phosphorylation, associated with a reduction in glucose uptake, and a switch to glutamine anaplerosis that boosted mitochondrial respiration and ROS production. Under conditions of glucose or glutamine withdrawal, this metabolic shift favored cell death. Whereas restoration of AKT signaling by expression of a constitutively active form of AKT rescued cell proliferation, it did not prevent starvation-induced death. In vivo, YAP/TAZ^low^ tumors survived due to the engagement of a compensatory lysosome-mediated activation of MAPK signaling. By combining YAP/TAZ and MEK inhibition, tumor growth durably regressed. YAP can also induce aerobic glycolysis through a direct interaction with HIF1α in a hypoxic environment [124, 125]. Therefore, the proglycolytic effect of YAP/TAZ engagement depends on their participation in various nuclear transcriptional complexes. In a muscle-derived UPS model, a combination of epigenetic modulators suppressed YAP1 activity and reduced sarcomagenesis through regulation of metabolism. In this case, YAP1 nuclear translocation was associated with a poor prognosis [126] and its inactivation by epigenetic modulators allowed the restoration of a clock gene-mediated unfolded protein response and muscle differentiation. It also promoted a switch toward lipid catabolism and autophagy, limiting YAP-driven UPS cell growth. The YAP/TAZ inhibitor Verteporfin is currently being tested in Ewing’s sarcoma (EWS) (Fig. 4 and Table 2).

#### Glutamine and arginine metabolic pathways contribute to STS growth

In a UPS mouse model harboring Kras mutations and p53 deletion in the muscle [59], tumors developed in the hindlimb and metastasized in the lung, as in the human disease. Additional deletion of HIF-2α or its binding partner aryl hydrocarbon receptor nuclear translocator (ARNT) enhanced tumor development. Use of an unbiased pan metabolomics strategy combining LC–MS and stable isotope metabolite tracing revealed a reliance on glutaminolysis for tumors, unlike muscle cells. Accordingly, glutaminase (GLS) inhibitors blocked UPS tumor growth in vivo and are the object of clinical trials in humans (Fig. 4 and Table 2). Mechanistically, GLS hydrolyses glutamine to glutamate, which is then dehydrogenated to alpha-ketoglutarate (αKG) by glutamate dehydrogenase GLUD or an aminotransferase (such as PSAT), boosting mitochondrial anaplerosis [127]. In this UPS model, tracing using C^13^- or N^15^-labeled glutamine demonstrated that glutamine is a carbon donor for the TCA cycle and a nitrogen donor for aspartate production from oxaloacetate. Aspartate is a crucial carbon source for purine and pyrimidine synthesis and sustains cell growth [128, 129]. Aspartate is also required for the conversion of citrullin into arginine through the activity of the rate-limiting argininosuccinate synthase 1 (ASS1) that initiates the urea cycle. This reaction generates arginine and contributes to the clearance of nitrogenous wastes. ASS1 deficiency has been observed in various cancers, including MFS, due to epigenetic silencing of its promoter [61]. Reexpression of ASS1 inhibited tumor growth and metastases. To investigate how arginine auxotrophy induced by ASS1 deficiency contributed to the progression of tumors, another study used a pegylated arginine deiminase (ADI-PEG20) to deplete arginine pharmacologically [89]. A short-term treatment by ADI-PEG20 applied to LMS cell lines, immediately induced cell proliferation arrest and autophagy. Upon prolonged therapy, cell lines became resistant to ADI-PEG20 due to the reexpression of ASS1 that regenerated arginine. A metabolomic profiling of treated cell lines revealed a reduction in PKM2 levels. In addition, U^13^C glucose tracing studies showed that carbons were shifted away from lactate and citrate production, and reoriented toward serine/glycine synthesis. Analysis of metabolic requirements for growth showed a reduced reliance on glucose and a reinforcement in OXPHOS and glutaminolysis, as an alternative source of TCA cycle intermediates via anaplerosis. In STS, a clinical trial using ADI-PEG20 in combination with Gemcitabine and Docetaxel has been launched (Fig. 4 and Table 2). Similarly, targeting glutamine metabolism through GLS inhibition could provoke the lethality of ASS1-deficient cancers and is currently being evaluated in GIST and NF1-mutated cancers (Fig. 4 and Table 2).

#### Complex metabolic rewiring in Kaposi’s sarcoma

Kaposi’s sarcoma (KS) is caused by a lytic oncogenic herpes virus (KSHV/HHV8), infecting endothelial cell precursors in immunosuppressed individuals. Infection, which is necessary but not sufficient for the growth of KS lesions, leads to the development of a vascular neoplasm associated with cytokine dysregulation driven by the virally encoded G protein coupled receptor (vGPCR). In lytically infected cells, vGPCR induced Rac1/NOX-dependent production of ROS that activated the redox sensitive STAT3 and HIF pathways [130]. Infected cells had increased lactate production and decreased mitochondrial respiration, a phenotype in part attributable to HIF1α activation [131]. Indeed, aerobic glycolysis favored by PKM2 induction sustains the maintenance of KS cells [132] (Fig. 3). Infected cells also exert a paracrine effect on neighboring endothelial cells. Indeed, PKM2 acts as a coactivator of HIF1α reinforcing the production of angiogenic cytokines [131]. Among them, PDGFRA, found phosphorylated in KS-biopsies [133], plays a major oncogenic role. HIF1α also participates in the reactivation of latently infected cells [134]. Upon transformation by KHSV, however, endothelial cells depended on glutamine for proliferation. KHSV also provoked an increase in ASS1 expression, in part through the action of KHSV-encoded miRNAs [62], leading to increased arginine production. Knockdown of ASS1 inhibited cell proliferation and iNOS-dependent, arginine-derived NO production. Treatment of KS cells with a NO donor-activated STAT3 without affecting ROS cell levels. A recent article questioned the relevance of these metabolic changes by comparing 2D versus 3D cultures of KHSV-infected cells [135]. An unbiased metabolomics analysis revealed significant changes in the levels of various non-essential amino acids in 3D cultures. GST-pull down studies showed that the viral K1 protein physically interacted with and activated the pyrroline-5-carboxylate reductase PYCR leading to increased proline production. This phenotype, abrogated by PYCR depletion, promoted 3D spheroid culture and tumorigenesis in nude mice. These results highlight the complex metabolic rewiring that occurs during infection and transformation by KHSV but also the need for appropriate in vitro culture systems to evaluate metabolic adaptation.

#### One carbon metabolism is overactive in aggressive STS

In Ewing’s sarcoma (EWS), the fusion protein resulting from a single translocation event between the regulatory domain of EWS and the DNA-binding domain of FLI1 behaves as a chimeric transcription factor called EF that enhances IGFR1 activation (Fig. 2A). Transcriptomic studies [60, 64] identified EF as an upstream regulator of PHGDH, PSAT, PSPH and SHMT1/2 genes involved in serine-glycine biosynthesis as well as SLC1A4/5 glutamine transporter genes (Fig. 3). Accordingly, knockdown of EF reduced the proportion of glucose-derived 3-phosphoglycerate reoriented toward serine and glycine synthesis; EWS cell lines were highly dependent on glutamine for growth and survival. Earlier work using a metabolomics approach with isotope labeling had already shown that a large proportion of glycolytic carbon was diverted into serine and glycine metabolism in melanoma; this was due to the amplification of the PHGDH gene [136], also elevated in high-risk EWS patients [100]. Serine or glycine can provide one carbon to tetrahydrofolate initiating the folate cycle. The enhancement of one-carbon metabolism, considered as an integrator of nutrient status [97], boosts the interconnected folate and methionine cycles leading to enhanced NADPH and nucleotide synthesis. NADPH regulates ROS-dependent death and methyl transfer contributes to epigenetic modifications. Knockdown of PHGDH recapitulated the effect of anti-metabolite chemotherapies and had a major effect on cell growth and epigenetic control. Two studies investigated the sensitizing effect of methionine restriction on chemo- or radio resistant models of RAS-driven colorectal cancer and STS, respectively [137, 138]. In the FSF Kras^G12D/+^; Tp53−/− STS mouse model, tumor development was triggered by the intramuscular injection of an adenovirus carrying the FlpO recombinase. In these aggressive tumors, only the combination of diet and radiation delayed tumor growth. By combining tumor metabolomics and metabolite tracing with a time-course analysis of data, alterations were observed for nucleotide and redox metabolisms. Interestingly, the consequences of methionine restriction could be detected at the metabolic level when applied to healthy individuals. These results indicated that a targeted dietary manipulation could improve tumor response to therapies.

#### Linking Wnt signaling alterations to metabolic rewiring

Physiologically**,** the canonical Wnt pathway participates to the maintenance of stem cell pools and cell fate, in part via the nuclear translocation of β-catenin, leading to its interaction with TCF/LEF transcription factors [139]. In a model of osteoblast differentiation, Wnt3a signaling induces aerobic glycolysis by increasing the level of glycolytic effectors (HK2, LDHA, PDK1, GLUT1). This process requires LRP5-mediated mTORC2/AKT activation but not β-catenin [140]. Other studies showed that Wnt engagement also reduced nuclear acetyl coenzyme A (AcCoA) levels and consequently impaired osteoblastic gene expression [141]. In contrast, in mature osteoblasts, Wnt-LRP5 boosted fatty acid oxidation and was required for bone mass increase [142]. In osteosarcoma (OS), Wnt participates in bone remodeling, maintenance of stem cell niches and EMT in collaboration with TGF-β and BMP signaling (reviewed in [143, 144]). In EWS, synovial sarcoma (SS), OS, and to a lesser extent in LMS [143, 145], a high level of Wnt activation, scored by the nuclear localization of β-catenin or LEF-1, is associated with a poor clinical outcome [46]. In OS, deletion of Wnt-related genes has been reported [146]. In some primary OS, the loss of the tumor suppressor RASSF1A enhanced Wnt activation through the AKT/GSK-3-Wnt/β-catenin pathway [147]. Other studies indicated that MEG3, a long non-coding RNA downregulated in OS, controlled the expression of several tumor suppressor genes and oncogenes including *P53*, *RB*, *MYC* and *TGF-β*; it also negatively regulated the expression of microRNA-184 (miR-184) and down-stream effectors of the Wnt/β-catenin pathway including β-catenin, TCF4 and c-MYC [148]. Therefore, downregulation of MEG3 attenuated its tumor suppressive effect and partly resulted in the upregulation of Wnt signaling. In this model, its impact on cell metabolism relied on mTORC1-mediated activation of the S6 kinase pathway and protein synthesis. In EWS, Wnt activation was also essential for the acquisition of a metastatic phenotype and controlled a proangiogenic switch via the secretion of specific extracellular matrix (ECM) proteins called angiomatrix in a TGF-β-dependent context [47]. However, overexpression of sFRP2, a secreted Wnt antagonist, promoted osteosarcoma invasion and metastatic potential [149]. Therefore, Wnt participates at various stages of STS progression.

### Loss of tumor suppressors affects several metabolic pathways

Tumor suppressors interrupt cell cycle and growth in a stressed environment, in part by regulating access to trophic pathways. Patients and mice carrying a hereditary defect in p53 (Li Fraumeni syndrome) or Rb1 (retinoblastoma) show a predisposition to sarcomas [49, 150]. In low/medium grade STS such as well-differentiated liposarcoma (WDLPS), the initial oncogenic event is the amplification of the p53 inhibitor MDM2. In DDLPS, MDM2 amplification synergizes with alterations affecting genes that regulate growth such as *CDK4* and *FRS2* [4], or that are required for adipocyte differentiation such as *JUN, DDIT3, PTPRQ, YAP1* or *CEBPA*, or with alterations of DNA methylation. More generally, in STS with complex genomes (LMS, UPS, MFS, LPS, MPNST), the accumulation of frequent somatic copy number alterations (SCNAs) and/or focal mutations of TSGs leads to the deregulation of the PI3K/AKT/mTOR axis, mitosis and chromosomal maintenance [48]. As in most cancers, the timing of occurrence of p53 mutations affects tumor progression and prognosis [8, 151–153]. Similarly, in mice, the combined loss of p53 [154] or CDKN2A (Ink4/Arf) [66] TSGs with oncogenic RAS lead to the development of undifferentiated STS. Conditional mutations in KRAS and p53 in muscle were sufficient to provoke high-grade STS with myofibroblastic differentiation [155].

As highlighted in Fig. 2A, p53 and AKT exert a negative feedback loop on each other, through PTEN and MDM2 regulation, respectively. Also, p53 indirectly counteracts AKT-dependent downstream effects on growth, apoptosis or metabolism [156, 157]. The tumor suppressive function of p53 depends on its role as transcription factor inducing cell cycle arrest or apoptosis via the CDKN1A (p21), or PUMA and NOXA effectors, respectively (Fig. 2A). However, in their absence, tumor suppression persists suggesting that additional mechanisms [158], including those with an impact on metabolism [156, 159] are also important (Fig. 3). Indeed, *GLUT* gene transcription is enhanced in STS-bearing *p53* mutations [160, 161]. p53 is anti-glycolytic partly through the induction of the expression of TIGAR and PARK2 regulators [162, 163]. TIGAR dephosphorylates fructose biphosphate (FBP) into fructose-6-phosphate (F6P), shifting glucose-6-phosphate (G6P) back toward the pentose phosphate pathway (PPP). Furthermore, the cytosolic form of p53 interacts with and inhibits G6P-dehydrogenase (G6PDH) by preventing the formation of the active dimer, therefore inhibiting PPP-dependent redox control and anabolism [164] (Fig. 3). Since TIGAR expression is not strictly p53-dependent, the resulting p53 effect may be difficult to predict with regards the engagement of the PPP, but it globally interferes with glycolysis. In STS, deep deletions or more frequently amplifications of TIGAR have been documented and high TIGAR expression correlates with a better outcome [50]. *PARK2/Parkin* is an E3 ubiquitin ligase regulating the degradation of mitochondrial proteins. It cooperates with the mitochondrial serine/threonine kinase PINK1 and contributes to mitochondrial fitness [163, 165]. p53-mediated mitochondrial homeostasis also involves the quality control of mitochondrial DNA (mtDNA) and the expression of cell death regulators [166]. Finally, p53 induces the expression of pyruvate decarboxylase (PDC) that regenerates mitochondrial oxaloacetate and reinitiates the TCA cycle, and that of isocitrate dehydrogenase 1 (IDH1) that converts cytosolic citrate into α-ketoglutarate. In a KRAS driven-PDAC model, p53-dependent accumulation of cytosolic α-ketoglutarate activates aKGDD enzymes that regulate 5-hydroxymethylcytosine-producing TET enzymes, allowing tumor cell differentiation and growth control [167] (Fig. 3).

### Metabolic fluxes and mitochondrial fitness in STS

Metabolite fluxes between organelles regulate the efficiency of various metabolic pathways in cells, but also contribute to the plasticity of metabolic adaptation.

#### Metabolic imbalances in STS

Through their evolution, most tumors tend to acquire metabolic features including an increase in nucleotide synthesis [168]. Investigations using PET-FDG uptake in STS patients confirmed the strong glycolytic bias documented in metastasized and poor prognosis STS [169, 170] such as ARMS [171] or ES [172]. However, these studies also revealed the considerable heterogeneity within a given tumor and between different tumor types, suggesting that the Warburg phenotype might be unstable and amenable to pharmacologic control [173]. Whereas the level of oxidative phosphorylation (OXPHOS) varies between tumors (Fig. 1), there is a general correlation between reduced mitochondrial activity, an epithelial-to-mesenchymal transition (EMT) gene signature and a poor prognosis [168]. Some STS tend to exhibit high levels of mitochondrial respiration compared to carcinomas (Fig. 1) [55, 174]. In vitro analysis of OS and RMS cell lines showed differences in the reliance on glycolysis versus respiration of tumors, with ARMS being in general less oxidative than OS or ERMS [175]. The equilibrium between glycolysis and mitochondrial respiration can be affected by various oncogenic alterations and/or metabolic requirements. Accordingly, the receptor tyrosine kinase Her4/ErbB4, an EGFR family member, is upregulated in several cancers including OS [176]. Exploration of xenograft models using untargeted metabolomics and 18F-FDG microPET/CT scan approaches showed that Her4 overexpression boosted glycolysis, glutaminolysis and OXPHOS in tumors. This hypermetabolic phenotype contributed to sustained growth and ATP production while conferring chemoresistance, as also shown in PDAC [177].

The crosstalk between metabolic pathways can also be altered in cancer as discussed in the following examples. Firstly, the upstream reaction committing glucose to glycolysis is catalyzed by phosphofructokinase-1 (PFK-1), itself allosterically inhibited by high ATP levels [178] (Fig. 3). Cancer cells express various PFK isoenzymes [179] such as the bifunctional 6-phosphofructo-2-kinase/fructose-2,6-bisphosphatase (PFKFB) that produces F2,6-BP, thereby overriding ATP-dependent inhibition of PFK1 [180, 181] (Fig. 3). Reciprocally, an activation of a F1,6-biphosphatase such as FBP1 enhances the gluconeogenic flow and restrains glycolysis [174]. The FBP2 isoform is frequently lost in STS including LPS, FS, LMS and UPS and lower FBP2 mRNA levels correlated with poor survival in LPS [55]. In the latter study, increasing FBP2 expression impaired sarcoma cell growth, through glycolysis inhibition and induction of mitochondrial biogenesis. The latter effect was due to FBP2 nuclear translocation where, independently of its enzymatic activity, it interacted with and inhibited c-Myc-driven transcriptional activation of TFAM, an inducer of mitochondrial biogenesis [182].

Secondly, the maintenance of glycolytic flow requires the regeneration of NAD+ which originates from cytosolic lactate dehydrogenase (LDH) activity and from the malate aspartate shuttle between mitochondria and cytosol [183] (Fig. 2). By regulating NAD+ levels, mitochondrial activity limits glycolysis and consequently the Warburg effect [184]. This suggests that the persistence of mitochondrial activity can be beneficial to tumors. In addition, an LDH activity has been identified in the mitochondria where it catalyzes the aerobic oxidation of lactate into pyruvate. It is thought to contribute to the maintenance or enhancement of OXPHOS in glycolytic cells [185, 186]. Pyruvate oxidation in the mitochondria depends on PDH activity, itself inhibited by PDK. The inhibition of PDK by dichloroacetate (DCA) shifts metabolism from glycolysis to glucose oxidation and boosts ROS production as well as mitochondria-dependent apoptosis in tumors [187]. This effect is exploited in EWS and other tumors where DCA synergizes with apoptosis-inducing drugs such as cisplatin. Manipulating ROS levels appears to be a promising therapeutic approach [188]. Indeed, scavenging mitochondrial ROS (mtROS) induces p53, reduces the cell transforming potential of oncogenic RAS and in some fibrosarcoma (FS) and RMS model cell lines suppresses tumor growth [17, 189].

Thirdly, the level of mitochondrial activity depends on the availability of coenzyme A (CoA) and the acetylated form, AcCoA. CoA synthesis requires the intracellular phosphorylation of pantothenate (or vitamin B5) by pantothenate kinases [190]. Reciprocally, pantothenate derives from the recycling of food-derived or cellular CoA through an extracellular degradative process involving the vanin pantetheinases [191, 192]. Interestingly, a high vanin1 (*VNN1*) level correlates with a better prognosis in STS patients [66]. Lack of Vnn1 in *CDKN2A* deficient mice enhanced the proportion of fibrosarcomas compared to that of other cancers. In RAS-driven mouse STS lines, Vnn1 exerted an anti-Warburg effect by enhancing CoA levels and mitochondrial activity to the detriment of glycolysis, and by maintaining cell differentiation.

#### Mitochondrial abnormalities disrupt the TCA cycle

Mitochondrial biogenesis depends on the transcriptional coactivator PGC1α [193]. This process regulates the transition from myoblast growth to differentiation and requires a switch from the classical to the alternative NF-kB activation pathway. The latter controls *PGC1α* transcription [194], in cooperation with MyoD [195]. In RMS and OS models, an alteration in this switch leads to the induction of the pro-glycolytic HK2 isoform through the persistent activation of the classical NF-kB pathway [196] (Fig. 3). This might also contribute to the incomplete mitochondrial biogenesis observed in a rat RMS model featuring a deficiency in respiratory potential and poor mtROS control, thereby enhancing tumorigenesis [197].

Mutations in TCA enzymes SDH [198] and FH [95], found in STS [57], are frequent in wild-type GIST without KIT or PDGFRA mutations [152]. They provoke an interruption of the TCA cycle, uncoupled from ATP production. Consequently, excess succinate diffuses in the cytoplasm where it inhibits aKGDD enzymes involved in the regulation of epigenetic modifications, DNA repair [199] or HIF degradation, rewiring cells toward glycolysis [200]. In a mouse ovarian cancer model, targeted knockdown of *Sdhb* resulted in enhanced proliferation and lead to a hypermethylated epigenome promoting EMT [198]. Using metabolic tracing and SeaHorse analysis, the authors documented an increased reliance on glutamine for cell survival and a reduced mitochondrial reserve capacity, rendering cells highly sensitive to the complex I inhibitor metformin.

Mutations in IDH1 and 2 lead to the production of 2-hydroxyglutarate [201], an inducer of HIF1α stabilization. *HIF1a* expression and hypoxia are associated with poor survival of sarcoma patients [68–70, 202–204]. Hypoxia regulates apoptosis resistance, cancer stemness, metastatic properties in RMS [71, 205] and is involved in ES, GIST and LPS progression [69, 70, 203, 204]. Uncoupling of electron transport chain (ETC) complexes from ATP production does not impede anaplerotic mitochondrial uptake of glutamine, transformed into glutamate via the activity of GLS to feed the reverse metabolic flow toward citrate production and anabolism [206] (Fig. 3). Accordingly, the growth of STS subtypes overexpressing GLS is sensitive to glutamine depletion in vitro and glutaminase inhibition in vivo [59].

Some components of the ETC are encoded by mtDNA. Therefore, alterations in mtDNA may lead to respiratory defects. In OS, insufficient or altered mtDNA is associated with stressed mitochondria and enhanced tumor invasiveness [207]. In an OS cell line, ethidium bromide induced-mtDNA depletion provoked a deficiency in cytochrome oxidase and OXPHOS, leading to enhanced glycolysis and EMT [208, 209]. Furthermore, mitochondrial dysfunction and loss of transmembrane potential provoked high cytosolic Ca^2+^ levels, triggering calcineurin-dependent mitochondria-to-nucleus retrograde signaling that resulted in AKT activation and glycolysis [210]. In a tunable model of mitochondrial dysfunction using cytoplasmic hybrids [57], impairment of respiration lead to NADH accumulation and cytosolic recycling into NAD+ by the malate deshydrogenase pathway. NAD+ boosted glycolysis and ATP-dependent cell migration. This suggests that glycolysis-derived ATP might be preferentially used during cell migration [211]. In conclusion, there is a high intra- and inter-tumor heterogeneity in mitochondrial activity, which can be enhanced or lost, depending on the tumor context.

### Tumor metabolome impacts STS progression

#### Metastasis

Aerobic glycolysis induced by oncogenic or hypoxic signaling provokes changes in the tumor metabolome. Lactate excretion, hypoxia-associated hypercapnia and acidification of the extracellular milieu accelerate the degradation of the extracellular matrix and facilitate metastasis [212, 213]. Reciprocally, as shown in STS [214, 215], cancer-associated fibroblasts can produce lactate and 3-hydroxybutyrate that boost cell growth, metastasis and angiogenesis when administered to tumor-bearing mice [216–218]. Lactate uptake by tumors feeds their oxidative metabolism [212, 216, 219] and requires the importer MCT1, a marker of mitochondrial activity and stemness in cancer and a target gene of the fusion protein ASPSCR1/TFE3 in alveolar soft part sarcoma (ASPS) [220]. The persistence of mitochondrial activity can enhance metabolic plasticity [221], mtROS-driven anoikis, metastasis [222, 223] or resistance to therapy as shown for LPS [224]. Metabolic plasticity, required during EMT transition [225], is still incompletely documented in poorly polarized and migration-prone mesenchymal tumor cells such as sarcoma cells [226]. Indeed, hybrid epithelial/mesenchymal (E/M) phenotypes or switching from E- to N-cadherin and vimentin expression contribute to aggressiveness, metastatic properties and drug resistance [226–229]. In addition, fusion protein events and translocations, frequent in childhood STS, can regulate epithelial differentiation [230, 231]. EMT is induced by cytokines such as FGFs, PDGF, TGF-β that enhance glycolysis and TCA activity [232]. TGF-β signaling synergizes with the AKT and NF-kB pathways, both potent drivers of glycolysis [233], but also antagonizes PDK4, thereby allowing pyruvate entry into the TCA. YB-1, an enhancer of HIF1α translation, is overexpressed in high-risk human sarcomas and promotes EMT and metastasis [234]. Hypoxia regulates the expression of several intracellular collagen-modifying enzymes, particularly OGDH enzymes that hydroxylate proline and lysine residues, contributing to the quality of collagen folding and the stiffness of the tissue, and thereby affecting cell migration [235]. In a UPS model, HIF1α enhances the expression of the intracellular enzyme procollagen-lysine, 2-oxoglutarate 5-dioxygenase 2 (PLOD2). Loss or overexpression of PLOD2 abrogates or restores, respectively, the metastatic potential of HIF1α-deficient tumors and human sarcomas show elevated HIF1α and PLOD2 expression in metastatic primary lesions [236]. Finally, HIF can enhance ECM degradation through the induction of various metalloproteases such as MMP or PLAUR, facilitating invasiveness.

#### Immunoreactivity

Several features including the level of infiltration of cytotoxic CD8+T cells or of myeloid cells, the expression of markers of immune-stimulation or -depression and the localization of these cells within the tumor, emerge as landmarks of tumor immunogenicity [8, 237–240]. STS display low mutational burden as compared to other cancer types and are generally considered to be poorly immunogenic and poorly responsive to immune checkpoint blockade [8, 241]. Synovial sarcoma, soft tissue and undifferentiated LMS are the three subtypes with the lowest CNV [8, 237] and display reduced immune infiltration, virtually devoid of lymphocytes [242–245]. In contrast, STS with several SCNAs, nucleotide and chromosome instabilities, such as undifferentiated LPS, MPNST [246–248], AS and GIST [243] and OS [244] can present high levels of lymphoid infiltration including CD8+ T cells. Consequently, STS display a wide range of immunophenotypes [238, 249, 250]. The metabolic rewiring imposed by tumors generates a situation of competition for essential energetic resources. This concerns glucose, vitamins and essential amino acids (serine, leucine, methionine, etc.) leading to impairment of immune cell functions and memory [19, 251, 252]. The exchange of fatty acids is required for the survival of immunosuppressive myeloid cells [253] or Tregs [254], particularly under conditions of activation of the PI3K/AKT/mTOR axis that boosts lipogenesis [255]. Other metabolites such as extracellular nucleotides released upon cell death can induce immunosuppression via various mechanisms [256–258]. Altogether, metabolic disturbances imposed by tumor cells directly contributes to the reorganization of the microenvironment but an exhaustive analysis of the immune landscape in STS is still lacking. Techniques such as Met-Flow [31] or SCENITH [32] should help dissecting the metabolic status of immunocytes.

## Conclusions

Unraveling the complexity of sarcoma genetics has benefited from the development and improvement of multi-omics strategies. The phenotypic and molecular description of genomic alterations can now be complemented with the identification of prognostic mechanistic signatures in patients. The heterogeneity and scarcity of STS originally limited the description of their metabolic landscapes but PET-FDG analysis has contributed to their staging, prognostication and evaluation of their response to therapy. Several cell-autonomous pathways or environmental factors influence the degree of conversion toward aerobic glycolysis, justifying the use of drugs that antagonize these processes. Nevertheless, the classic distinctions between glycolytic and oxidative tumors must be carefully reconsidered; hybrid phenotypes may confer more adaptable behaviors to STS cells. Several important metabolic pathways associated with STS progression such as those of one carbon and arginine metabolisms, or the PGI pathway might provide novel therapeutic options in combination with conventional therapies. In addition, the development of novel animal models or 2D/3D culture systems has highlighted the metabolic plasticity of these tumors that may impact their energetic resources. However, these adaptations may have a price, rendering tumor cells more sensitive to combined therapies.

Oncogenic signals can lead to the expression of isoforms of glycolytic enzymes that display new functions tilting the balance between glycolysis and mitochondrial activity. Similarly, p53 can also act as a regulator of G6PDH activity, impacting biosynthetic pathways. Some metabolites such as α-ketoglutarate have emerged as key effectors of p53 action, whereas others behave as oncometabolites leading to alterations of genome integrity, metastatic behavior and therapeutic response. The balance between glycolysis, glutaminolysis and OXPHOS depends on the respective availability of key metabolites, such as amino acids, NAD+/NADH, lactate or VitB5, that regulate STS progression or differentiation. Metabolomic studies have already shown that novel metabolite signatures will complement conventional biomarkers and help stratifying prognosis and therapeutic options. The stability of the glycolytic phenotype also depends on mitochondrial activity. Alterations of mitochondrial fitness observed in STS upon alterations of mtDNA or TCA enzymes aggravate the prognostic of tumors or can affect their chemoresistance. An unstable tumor metabolome has tumor-intrinsic or extrinsic effects causing it to be pro-metastatic or immunosuppressive. Therefore, the combination of drugs targeting different metabolic pathways should impact both tumor and immune cells in a concerted manner to reinvigorate anti-tumor immunity while tilting the balance toward cell differentiation over growth.

## Data Availability

Data used for the bioinformatics analysis come from the publically available TCGA database. The code to reproduce the analysis is available at GitHub (https://github.com/guillaumecharbonnier/mw-miallot2021).

## Abbreviations

3HB: 3‐Hydroxybutyrate
AcCoA: Acetyl coenzyme A
AIDS: Acquired immunodeficiency syndrome
aKGDD: Alpha-ketoglutarate dioxygenase
ARMS: Alveolar rhabdomyosarcoma
AS: Angiosarcoma
ASPS: Alveolar soft part sarcoma
ATF6: Activating transcription factor 6
ATRX: α-Thalassemia/mental retardation syndrome x-linked
BCL2: B-cell cell/lymphoma 2
BCL-XL: B-cell lymphoma-extra large
BIM: BCL-2-interacting mediator of cell death
BLCA: Bladder urothelial carcinoma
BMP: Bone morphogenetic proteins
BRCA: Breast invasive carcinoma
CAF: Cancer-associated fibroblast
CDK4: Cyclin-dependent kinase 4
CDKN1A = p21: Cyclin-dependent kinase inhibitor 1a
CDKN2A = p16: Cyclin-dependent kinase inhibitor 2a
CEBPA: CCAAT/enhancer-binding protein alpha
CNV: Copy number variations
CS: Canine sarcoma
DCA: Dichloroacetate
DDIT3: DNA damage-inducible transcript 3
DDLPS: Dedifferentiated liposarcoma
DNMT1: DNA methyltransferase 1
ECM: Extracellular matrix
EF: EWS/FLI1 fusion protein
EGR1: Early growth response protein 1
EMT: Epithelial to mesenchymal transition
ERMS: Embryonal rhabdomyosarcoma
ESCA: Esophageal carcinoma
ETC: Electron transport chain
EWS: Ewing sarcoma
F2,6BP: Fructose-2,6-bisphosphate
FASL: Fas ligand
FBP: Fructose-biphosphate
FGFs: Fibroblast growth factors
FLI1: Friend leukemia integration 1 transcription factor
FRS2: Fibroblast growth factor receptor substrate 2
FS: Fibrosarcomas
FUS/DDIT3: Fused in sarcoma (FUS) to DNA damage inducible transcript 3 (DDIT3)
G6P: Glucose-6-phosphate
GFR: Growth factor receptor
GIST: Gastrointestinal stromal tumor
GLUT: Glucose transporter
GSK: Glycogen synthase kinase
GTPase: Guanosine triphosphate hydrolase
HHV8: Human herpes virus 8
HIF: Hypoxia inducible factor
HIPPO: Hippopotamus (according to the tissue overgrowth)
HK: Hexokinase
IAP: Inhibitors of apoptosis proteins
IDH1: Isocitrate dehydrogenase 1
IGF1: Insulin-like growth factor 1
IGF1R: Insulin-like growth factor 1 receptor
KRAS: Kirsten rat sarcoma viral
KS: Kaposi sarcoma
KSHV: Kaposi sarcoma-associated herpesvirus
LDH: Lactate deshydrogenase
LEF-1: Lymphoid enhancer-binding factor 1
LIHC: Liver hepatocellular carcinoma
LMS: Leiomyosarcoma
lncRNA: Long non-coding RNA
LPR5: Low-density lipoprotein receptor-related protein 5
LPS: Liposarcoma
LUAD: Lung adenocarcinoma
LUSC: Lung squamous cell carcinoma
MAPK: Mitogen-activated protein kinase
MCA: Methylcholanthrene
MCL1: Myeloid leukemia cell differentiation protein
MCT: Monocarboxylate transporter
MDH: Malate dehydrogenase
MDM2: Mouse double minute 2 homolog
MEG3: Maternally expressed 3
MEK: Mitogen-activated protein kinase
MESO: Mesothelioma
MFS: Myofibrosarcoma
MFH: Malignant fibrous histiocytoma (equivalent to UPS)
miR: Micro RNA
mitROS: Mitochondrial reactive oxygen species
MLS: Myxoid liposarcoma
MMP: Matrix metallopeptidases
MPNST: Malignant peripheral nerve sheath tumors
MSC: Mesenchymal stem cells
mtDNA: Mitochondrial DNA
mTOR: Mammalian target of rapamycin
mTORC1: Mammalian target of rapamycin complex 1
MyoD: Myoblast determination protein 1
NAD: Nicotinamide adenine dinucleotide
NADPH: Nicotinamide adenine dinucleotide phosphate
ncRNA: Non-coding RNA
NF: Neurofibromin
NFκB: Nuclear factor kappa-light-chain-enhancer of activated b
NOX: NADPH oxidase
NOXA: Phorbol-12-myristate-13-acetate-induced protein 1
OAA: Oxaloacetate
OS: Osteosarcoma
OVCA: Ovarian serous cystadenocarcinoma
OXPHOS: Oxidative phosphorylation
PARK2: Phosphatidic acid-regulated protein kinase
PAAD: Pancreatic adenocarcinoma
PDAC: Pancreatic ductal adenocarcinoma
PDC: Pyruvate decarboxylase
PDGF: Platelet-derived growth factor receptors
PDHK: Pyruvate dehydrogenase kinase
PDK: 3-Phosphoinositide-dependent protein kinase
PERK: Protein kinase r-like endoplasmic reticulum kinase
PET-FDG: Fluorine-18-fluorodeoxyglucose positron emission tomography
PFK: Phosphofructokinase
PFKFB: 6-Phosphofructo-2-kinase/fructose-2,6-biphosphatase
PGC1a: Peroxisome proliferator-activated receptor gamma coactivator 1-alpha
PGK1: Phosphoglycerate kinase 1
PHGDH: 3-Phosphoglycerate dehydrogenase
PI3K: Phosphatidylinositol-3-kinase
PKM2: Pyruvate kinase M2
PLAUR: Plasminogen activator, urokinase receptor
PLOD2: Procollagen-lysine,2-oxoglutarate 5-dioxygenase 2
PPP: Pentose phosphate pathway
PSAT: Phosphohydroxythreonine aminotransferase
PSPH: Phosphoserine phosphatase
PTEN: Phosphatase and tensin homolog
PTPRQ: Protein tyrosine phosphatase receptor type q
RAC1: Phosphoserine phosphatase
RAS: Rat sarcoma oncogene
RASSF14: Ras-association domain family 1 isoform a
Rb1: Retinoblastoma protein
RMS: Rhabdomyosarcoma
ROS: Reactive oxygen species
RREB1: Ras-responsive element binding protein 1
S6K: S6 kinase
SCNAs: Somatic copy-number alterations
SHMT1/2: Serine hydroxymethyltransferase 1 and 2
SMARCB1: SWI/SNF-related, matrix-associated, actin-dependent regulator of chromatin, subfamily b, member 1
SS: Synovial sarcoma
STAD: Stomach adenocarcinoma
STS: Soft tissue sarcoma
SV: Structural variation
TA: Transit amplifying
TAZ: Transcriptional coactivator with PDZ-binding motif
TCA: Tricarboxylic acid cycle
TCF: T-cell factor
TEAD: Tea domain family member 1
TERT: Telomerase reverse transcriptase
TF: Transcription factor
TFAM: Transcription factor a mitochondrial
TGF-β: Transforming growth factor beta
TIGAR: p53-induced glycolysis regulatory phosphatase
P53: Tumor protein p53
TRAIL: TNF-related apoptosis-inducing ligand
TSC1/2: Tuberous sclerosis complex 1/2
TSG: Tumor suppressor gene
UPR: Unfolded protein response
UPS: Undifferentiated pleomorphic sarcoma
VEGF: Vascular endothelial growth factor
vGPCR: Viral encoded g protein-coupled receptor
VHL: Von Hippel–Lindau tumor suppressor
VNN1: Vanin-1
WDLS: Well-differentiated liposarcoma
WNT: Wingless/integrated
YAP1: Yes-associated protein
YB-1: Y-box binding protein 1
